# Genetic and epigenetic factors affect RET gene expression in breast cancer cell lines and influence survival in patients

**DOI:** 10.18632/oncotarget.8417

**Published:** 2016-03-28

**Authors:** Paola Griseri, Ornella Garrone, Alessandra Lo Sardo, Martino Monteverde, Marta Rusmini, Federica Tonissi, Marco Merlano, Paolo Bruzzi, Cristiana Lo Nigro, Isabella Ceccherini

**Affiliations:** ^1^ UOC Medical Genetics, IRCCS Giannina Gaslini Institute, Genoa, Italy; ^2^ Unit of Medical Oncology, Department of Oncology, S. Croce & Carle Teaching Hospital, Cuneo, Italy; ^3^ Laboratory of Cancer Genetics and Translational Oncology, Department of Oncology, S. Croce & Carle Teaching Hospital, Cuneo, Italy; ^4^ Clinical Epidemiology, IRCCS AUO San Martino IST, Genoa, Italy

**Keywords:** RET gene, breast cancer, single nucleotide polymorphism, gene expression, resistance to hormonal therapy

## Abstract

Germline and somatic mutations play a crucial role in breast cancer (BC), driving the initiation, progression, response to therapy and outcome of the disease. Hormonal therapy is limited to patients with tumors expressing steroid hormone receptors, such as estrogen receptor (ER), nevertheless resistance often limits its success.

The *RET* gene is known to be involved in neurocristopathies such as Hirschsprung disease and Multiple Endocrine Neoplasia type 2, in the presence of loss-of-function and gain-of-function mutations, respectively. More recently, *RET* over-expression has emerged as a new player in ER-positive (ER+) BC, and as a potential target to enhance sensitivity and avoid resistance to tamoxifen therapy.

Therefore, targeting the RET pathway may lead to new therapies in ER+ BC. To this end, we have investigated the molecular mechanisms which underlie *RET* overexpression and its possible modulation in two BC cell lines, MCF7 and T47D, showing different *RET* expression levels. Moreover, we have carried out a pilot association study in 93 ER+ BC patients. Consistent with the adverse role of *RET* over-expression in BC, increased overall survival was observed in carriers of the variant allele of SNP rs2435357, a *RET* polymorphism already known to be associated with reduced *RET* expression.

## INTRODUCTION

Breast cancer (BC) is the leading cause of cancer death in females with an incidence growing worldwide [1,2]. Like other cancers, BC is considered as a complex disease in which gene aberrations, cellular context and environmental influences concur to tumor initiation and progression. Germline and somatic gene mutations play a crucial role in BC, being involved in inherited cancer syndromes [3,4], associated with either specific morphological stages [5] or response to therapy and poor disease outcome [6–8]. The therapeutic approach for women with BC includes a combination of chemotherapy, targeted and hormonal therapy. The latter is limited to patients with tumors expressing steroid hormone receptors, such as estrogen receptors (ER) and progesterone receptors (PR), which are about 65% of total cases [9–11]. The use of selective estrogen receptor modulators (SERMs), such as tamoxifen or aromatase inhibitors (AI), has led to remarkable improvements in cure efficacy for ER-positive (ER+) BC [12]. Nevertheless *de novo* and acquired resistance often limits the success of this therapeutic strategy in BC patients [13–15]. Understanding the molecular basis of such resistance may help us to develop alternative therapeutic strategies, thus improving response to treatment and preventing cancer deaths.

In this scenario, the receptor tyrosine kinase *RET* (REarranged during Transfection) has emerged as a new player in ER+ cancer development as well as a potential target to enhance sensitivity of BC to tamoxifen therapy and to avoid tamoxifen resistance [16]. *RET* activation is secondary to the formation of a multi-protein complex, including one of four soluble ligands, namely Glial cell Derived Neurotrophic Factor (GDNF), Neurturin (NRTN), Artemin (ARTN) or Persephin (PSPN), and one of four GPI-linked co-receptors (GFRα1-4), that leads to RET dimerization and autophosphorylation of tyrosine residues in the intracellular tyrosine kinase domain [17,18]. Alternative splicing of transcripts encoding the *RET* receptor leads to the isoforms RET9 and RET51 (either 9 or 51 carboxy-terminal aminoacids, respectively) with distinct biological functions [19–21].

Germline mutations of the *RET* gene are responsible for two different genetic disorders, Hirschsprung's disease (HSCR) and Multiple Endocrine Neoplasia type 2 (MEN2), due to loss-of-function and gain-of-function mutations, respectively. Somatic mutations of the *RET* gene are also responsible for sporadic Medullary Thyroid Carcinoma (MTC), while gene rearrangements have been found in papillary thyroid carcinoma (PTC) and recently identified in lung adenocarcinoma [22]. Several studies have investigated the role of *RET* common SNPs in *RET*-related diseases [23–26]. Interestingly, one SNP (rs2435357) located in an intronic enhancer, was shown to reduce *RET* expression and therefore inversely associate with HSCR disease and sporadic MTC [26,27].

Several independent studies recently identified *RET* as a key player in BC pathogenesis [16,22]. In particular, *RET* and *Gfra1* were over-expressed in a subset of ER+ tumors [28–30] and elevated *RET* levels correlated with decreased metastasis-free survival [31,32].

Estrogens induce transcription of *RET* and other ER+ dependent genes [33,34], in ER+ BC cell lines such as MCF7 and T47D [16,35,36], by activating the estrogen receptor. Regulatory elements responsive to this latter receptor are located at -50kb and + 32kb from the *RET* gene [35,36]. Moreover, additional target elements are located in the *RET* locus [37,38]. *RET* expression is also dependent on chromatin structure [39] and histone deacetylase (HDAC) inhibitors, such as Tricostatin A, Sodium Butyrate (Nabut), and 5 aza-2′ deoxycytidine (deAza), have opposite effects on estrogen mediated transcription in BC cell lines, thus affecting anti-estrogen therapy [40]. In particular, in ER-negative (ER-) cell lines, treatment with HDAC inhibitors reactivated *ER* expression and cellular response to hormone therapy [41,42] while in ER+ cell lines the same drugs have been associated with transcriptional down regulation of *ER* mRNA and its responsive genes [43,44]. Both *RET* and *ER* are tightly connected and drive proliferation and cell survival in luminal BC, thus demonstrating the potentiality of targeting both pathways [38]. Indeed, *RET* inhibition was shown to increase the efficacy of antiestrogen drugs, and combined therapy of tamoxifen with vandetanib was proven as a potential treatment strategy for *RET* positive luminal breast cancers [45].

Besides the expression of RET in ER+ BC, data suggest that RET can be expressed at low levels also in ER-ve and triple negative tumors. TFAP2C has been shown to induce ER independent *RET* expression, with important implications for the ER-ve breast cancers, such as MDA-MB-453 [38].

Finally, Morandi et al. [46] reported that the *RET-GDNF* pathway is a key determinant of response and resistance to AI in ER+ tumors.

Based on these lines of evidence, *RET* over-expression in BC might be due to transcriptional mechanisms involving estrogen receptors and chromatin conformation, so that targeting the *RET* pathway may lead to the development of new therapies in ER+ BC. To this end, we have investigated the molecular mechanisms (either genomic, transcriptional or post transcriptional) which underlie *RET* over-expression and its possible modulation in BC. Looking for *RET* genetic variants responsible for *RET* expression modulation, as well as BC progression and outcome, we also carried out a pilot association study in 93 ER+ BC patients.

## RESULTS

### Differential expression of *RET*, co-receptors and ligands in MCF7 and T47D

**Figure 1:**
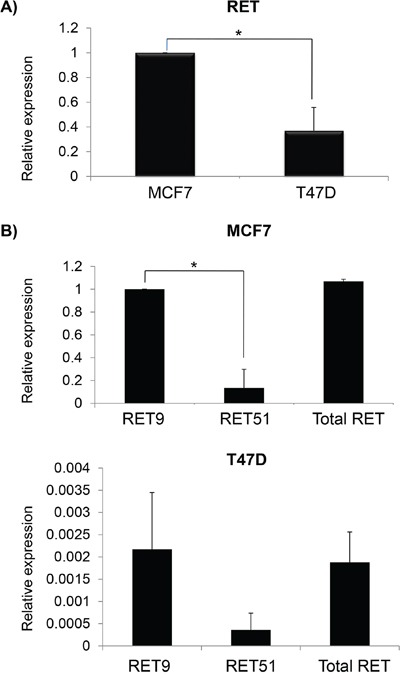
Differential expression of total *RET* and its isoforms in breast cancer cell lines **A.** Relative basal RET mRNA levels in MCF7 (equal to 1) and T47D. **B.** mRNA quantification of *RET9* and *RET51* isoforms in MCF7 (top) and in T47D (bottom). Values are the mean ±SD of at least two independent experiments performed in triplicate; asterisks (*) indicate statistically significant differences (*p<0.05; **p<0.01).

We have quantified *RET* mRNA levels in MCF7 and T47D in order to confirm that these cell lines, originated from ER+ BC, express different amounts of the *RET* gene. In agreement with literature data, we found that MCF7 showed significantly higher levels of *RET* compared to T47D (Figure 1A) [30,36]. Since the two isoforms *RET9* and *RET51* exert different oncogenic properties and differ for their C-terminals, we quantified and compared their relative amounts. To discriminate between *RET9*, *RET51* and total *RET*, we set up two amplicons with a same forward primer, located upstream of the C-tail, and two alternative distal reverse primers, each specific for one isoform, in addition to an internal control amplimer in a 5′ region common to both isoforms. We found that MCF7 express a greater amount of the two isoforms than T47D (compare Y-axis scale in Figure 1B). Both cell lines express more *RET9* than *RET51*, and in MCF7 this difference is statistically significant (Figure 1B). In addition to *RET*, we also investigated the mRNA levels of *RET* ligands (*GDNF*, *NRTN*, *ARTN* and *PSPN*) and co-receptors (*GFRa1-3*), except *GFRa4* which seems to be expressed only in thyrocytes [18]. The results obtained are shown in Table 1. Consistent with literature data, MCF7 cells express very low levels of *GDNF*, which is undetectable in T47D [29]. The other genes display similar pattern of expression in the two cell lines, with the only exception of *GFRa1*, which is mainly express in MCF7 but not in T47D, and *GFRA2* which is predominant in T47D, but poorly expressed in MCF7.

**Table 1:** Expression of members of the *RET* pathway in breast cancer cell lines: qualitative analysis of *RET*, its ligands and co-receptors in MCF7 and T47D

Genes	MCF7	T47D
*RET*	+++	+
*GDNF*	+/−	-
*NRTN*	++	++
*ARTN*	++	+++
*PSPN*	++	+++
*GFRA1*	+	-
*GFRA2*	-	+
*GFRA3*	+/−	+/−

### *RET* over-expression in MCF7 is due to transcriptional mechanisms

**Figure 2:**
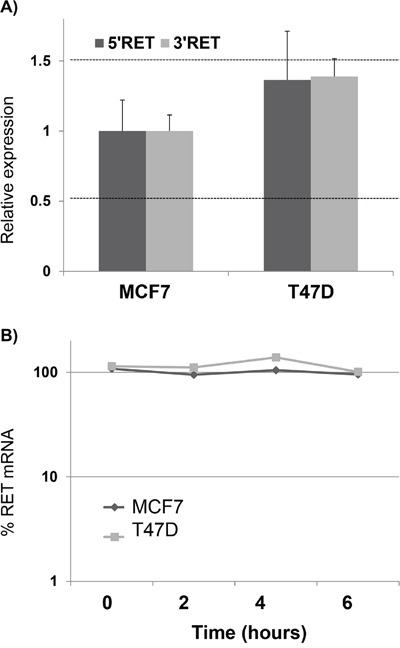
*RET* over-expression in MCF7 is due to transcriptional mechanisms **A.** Quantification of *RET* genomic content in MCF7 and T47D by using two amplimers designed in the 5′ and 3′ UTRs respectively. Thresholds to detect DNA deletion or duplication were settled to 0.5 and 1.5 of % genomic DNA compared to a reference genome, respectively. **B.** Quantification of *RET* mRNA half-life by DRB pulse chase experiments in MCF7 and T47D. Log-linear regression curves are shown. Values are the mean ±SD of at least two independent experiments performed in triplicate.

We investigated the mechanisms potentially underlying the different expression of *RET* in MCF7 and T47D cell lines. First of all, to exclude duplications in MCF7 or deletions in T47D genomes, possibly accounting for their different *RET* mRNA levels, we quantified the relative *RET* locus dosage in the two BC cell lines. To this end, we developed a real time qPCR from genomic DNA, designing two amplimers in the 5′ and 3′ end of the *RET* gene respectively, and setting the threshold for RET amount compared to a reference gene at <0.5 for deletions and >1.5 for duplications. Consistent with known data, the results clearly showed a comparable dose of *RET* DNA in MCF7 and T47D (Figure 2A). Then, we sought to see whether the difference observed in *RET* mRNA levels between the two cell lines was due to transcriptional or post-transcriptional mechanisms. To this end, we investigated *RET* mRNA half-life in the two cell lines. Since preliminary experiments had suggested that *RET* mRNA is very stable, we decided to quantify *RET* mRNA amounts at 0, 2, 4 and 6 hours after transcriptional inhibition with DRB treatment. As shown in Figure 2B, *RET* half-life has turned out to last more than 6 hours in both cell lines, and even longer in the T47D cells that, having less *RET* transcript than MCF7, and presumably a less stable mRNA, were treated with DRB for up to 12 hours without observing any significant difference in the *RET* mRNA level (Supplementary Figure S1). These data prompted us to hypothesize that the differences observed in *RET* mRNA levels in MCF7 and T47D might be due mainly to transcriptional mechanisms.

### Estrogen enhanced expression of *RET* and *Artemin* in breast cancer cells

**Figure 3:**
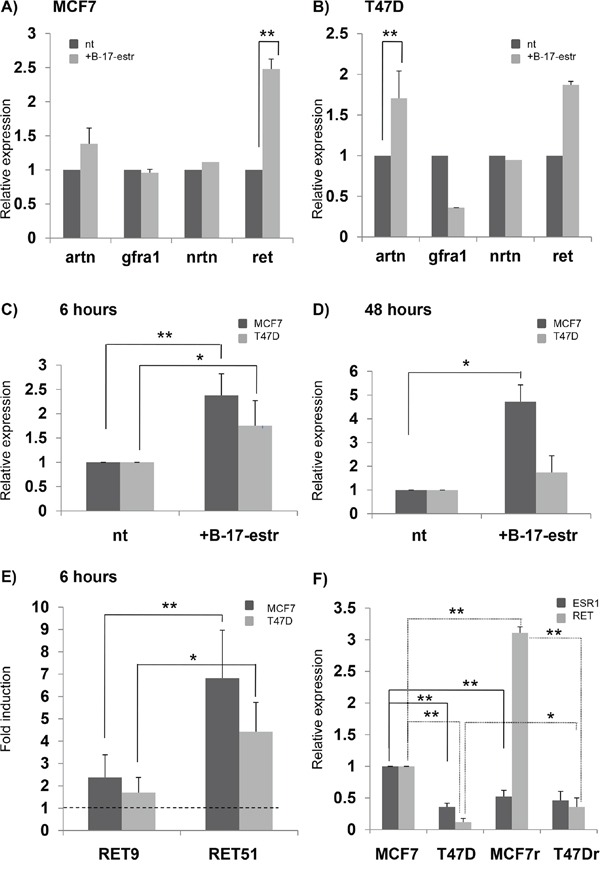
Effect of estrogens on *RET*-pathway gene expression mRNA quantification of *RET*, *GFRa1*, *NRTN* and *ARTN* after 6 hours of 17β-estradiol (B-17-estr) treatment in MCF7 **A.** and T47D **B.** *RET* expression as assessed after 6 hours **C.** and 48 hours **D.** of B-17-estr treatment in both the MCF7 and T47D cell lines. **E.** *RET9* and *RET51* mRNA fold induction after 6 hours of B-17-estr administration in MCF7 and T47D. **F.** Levels of *RET* and estrogen receptor (*ESR1*) mRNAs induced by either estrogens depleted DMEM media (MCF7 and T47D) or standard DMEM (MCF7r and T47Dr). Values are the mean ±SD of at least two independent experiments performed in triplicate; asterisks (*) indicate statistically significant differences (*p<0.05; **p<0.01).

To evaluate whether and how the expression of the *RET* gene and its co-receptors and ligands depends on estrogen levels in BC cell lines, we treated both MCF7 and T47D with 17-β-estradiol for 6 and 24 hours and performed a realtime PCR for *RET*, *GFRa1*, *ARTN*, and *NRTN* genes. We found that after 6 hours only *RET* and *ARTN* showed statistically significant differences in mRNA levels between treated and untreated samples (Figures 3A and 3B). This is consistent with previous observations showing that not only *RET* but also *ARTN* is an estrogen inducible gene and plays a role in BC pathogenesis [35,36,47]. At 6 hours, β-estradiol increased *RET* expression both in MCF7 and T47D, while its effect disappeared in T47D after 48 hours (Figures 3C and 3D). Interestingly, the amount of *RET9* and *RET51* mRNA, quantified in the two cell lines after the estrogen treatment, displayed a greater fold induction for *RET51* than *RET9*. These data are in agreement with an already reported different response to estrogens of the two isoforms (Figure 3E) [37]. To confirm the effect of estrogens on *RET* expression, we compared levels of *RET* mRNA induced in MCF7 and T47D cell lines by two different cell culture media: standard DMEM and estrogens-depleted DMEM media, this latter lacking of phenol-red. As shown in Figure 3F, estrogens, provided either specifically or by means of standard DMEM medium, did activate *RET* expression. In order to assess whether *RET* transcript levels do correlate with ER amount, we then compared *RET* expression levels with Estrogen Receptor alpha (*ESR1*) mRNA levels in MCF7 and T47D. After growing cells in either normal DMEM medium or estrogens depleted DMEM medium, no correlation was demonstrated between *RET* and *ESR1* expression (data not shown).

### Inflammatory stimuli which may affect *RET* transcription

**Figure 4:**
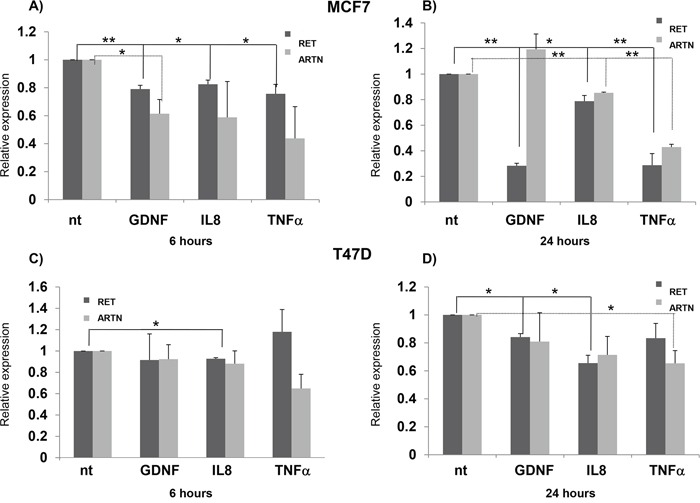
Inflammatory stimuli and *RET* transcription *RET* and *ARTN* expression in MCF7 cells treated for 6 hours **A.** and 24 hours **B.** with GDNF/gfra1, IL8 and TNFalpha. Same experiments repeated in T47D cells are reported in panels **C.** and **D.** Values are the mean ±SD of at least two independent experiments performed in triplicate; asterisks (*) indicate statistically significant differences (*p<0.05; **p<0.01).

Considering the well-known connection between tumors and immunity, and the involvement of the *RET* gene in the immune system development and function [48], we treated BC cell lines for 6 and 24 hours with inflammatory stimuli which could modulate *RET* expression, namely the RET ligand GDNF, IL8, and TNFα. In particular, we tested the expression levels of both *ARTN* and *RET*, two genes demonstrated above to have an estrogen-dependent expression and considered to be oncogenic in BC cells. As shown in Figure 4, these treatments induced different effects in MCF7 and T47D cells. A significant decrease in *RET* expression with all the treatments (GDNF/GRFRA1, IL8, TNFα), was observed at both 6 and 24 hours in MCF7. Conversely, *ARTN* significantly decreased after 6 hours with GDNF treatment (Figure 4A), and after 24 hours with IL8 and TNFα treatments (Figure 4B). In T47D, RET was down-regulated by IL8, after both 6 and 24 hours of treatment, and by GDNF, after 24 hours. No change in *ARTN* levels was detected after 6 hours in any condition, while *ARTN* levels decreased after 24 hours of TNFα treatment (Figures 4C and 4D). Overall these data suggest that IL8, and at lesser extent TNFα, is able to induce a down-regulation of *RET* expression. In line with previous observations about IL-8 upregulation in the presence of GDNF+GFRα1 in SK-N-MC neuroectodermal tumor cells, stably transfected with the human *RET* gene, as well as in TT medullary thyroid carcinoma cells and PTC-1 papillary thyroid carcinoma cells [49,50], we sought to verify whether RET could induce IL-8 also in BC cell lines. We found the mRNA levels of *IL-8* were significantly increased after 6 hours incubation of MCF7 but not of T47D with GDNF plus GFRα1, while incubation of 24 hours did not induce any change in both these BC cell lines (Supplementary file and Supplementary Figure S2).

### Chromatin structure and *RET* expression

**Figure 5:**
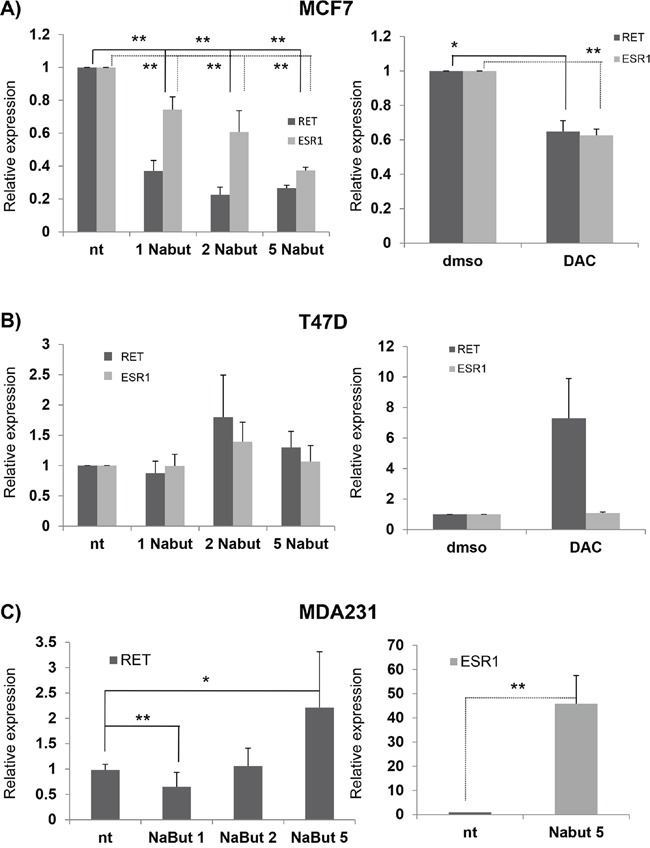
HDAC inhibitors and *RET* expression in breast cancer cell lines mRNA expression of *RET* and *ESR1* in MCF7 **A.** and T47D **B.** cells after treatment with 1, 2, 5 nM Nabut (left panel, normalised to untreated cells) and 10uM 5-Aza-2′-deoxycytidine (DAC) (right panel, normalised to DMSO treated cells). *RET* and *ESR1* expression levels in MDA231 upon Nabut treatments are reported in the left and right C panels respectively. Values are the mean ±SD of at least two independent experiments performed in triplicate; asterisks (*) indicate statistically significant differences (*p<0.05; **p<0.01).

In order to verify whether and how *RET* transcription is modulated by HDAC inhibitors in BC, we treated the above cell lines with Nabut at different concentrations (1, 2 and 5 nM) and investigated, 48 hrs later, the levels of *RET* and *ESR1* mRNA thus induced. As shown in Figure 5A (left panel), in MCF7 we observed a Nabut dose-dependent, statistically significant down-regulation of *RET* and *ESR1* compared to untreated cells, with the trend of the two genes clearly correlated (R=0.847). Surprisingly, T47D cells were not affected by the drug (Figure 5B, left panel). Similar results were observed after treatments with 5-Aza-2′-deoxycytidine (DAC): in MCF7 both *RET* and *ESR1* decreased, while in T47D we observed an increase in *RET* mRNA levels which is statistically significant (Figures 5A and 5B, right panels). Overall, these data suggest that in our cellular model *RET* expression is affected by the chromatin structure.

**Figure 6:**
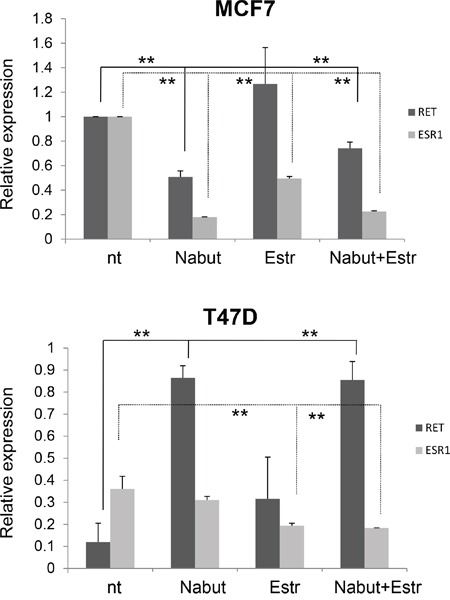
Combined effect of HDAC inhibitors and estrogens Effect of 48 hours treatment with Nabut and 17-β-estradiol on *RET* and *ERS1* transcript levels in MCF7 (top panel) and T47D (bottom panel) cell lines. A different scale has been used to optimize visualization of the fold change of *RET* expression in the two cell lines. Values are the mean ±SD of at least two independent experiments performed in triplicate; asterisks(*) indicate statistically significant differences (*p<0.05; **p<0.01).

A similar experiment has already been carried out in the ER- cell line MDA231, physiologically expressing very low levels of *RET* [36]. After Nabut treatments, and compared to untreated cells, we observed a dose-dependent increase of *RET* expression (Figure 5C, left panel) while the *ESR1* mRNA levels resulted significantly enhanced after Nabut 5nM, but undetectable for lower concentrations (Figure 5C, right panel). In order to identify the treatment most effectively affecting *RET* expression, we treated both MCF7 and T47D with 17-β-estradiol, Nabut and the two drugs together for 48 hours. These treatments confirmed the opposite effects of the two compounds on MCF7 and T47D (Figure 6). Moreover, when we treated the cells with Nabut and β-estradiol together, we observed a *RET* expression more comparable to the effect of Nabut alone than β-estradiol in both MCF7 (Figure 6, top panel) and T47D (Figure 6, bottom panel) cell lines, demonstrating the upstream effect of Nabut with respect to estrogens.

### *RET* genotyping and statistical analysis and clinical study

**Figure 7:**
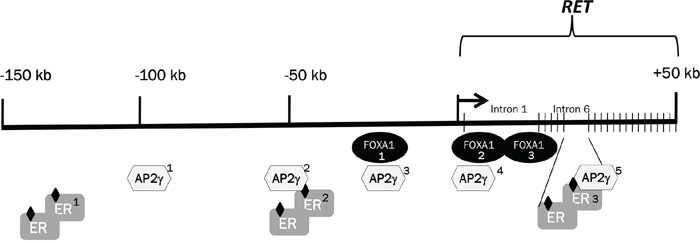
Selected transcription factors and positional map of their binding sites at the *RET* locus Schematic representation of the 150 kb upstream of the *RET* gene followed by the 50 kb of the *RET* genomic region. The localization of ER and FOXA1 candidate binding regions has been obtained from the UCSC browser, while the five TFAp2C positions were published by Tan et al. [37]

The different *RET* expression modulation assessed in the MCF7 and T47D cell lines in response to Nabut and estrogens may be accounted for by their *RET* gene sequence, including functional elements lying in the *RET* regulatory regions. As two ER-responsive enhancers (ERE) have already been located at -50 kb from the transcription start and in intron 6 of the *RET* gene [35], we looked up the UCSC genome browser and performed a bioinformatic analysis of the *RET* locus. Results thus obtained and data already known were used to achieve a refined map of ER-sensitive loci at the *RET* locus (Figure 7). We sequenced genomic DNA samples from MCF7 and T47D cells for all the above mentioned putative ER-responsive regions, as well as a conserved region in intron 1, which is known to act as a *RET* enhancer during development [24,51]. Only variants at two single nucleotide polymorphisms, rs12247450 and rs2435357, resulted to differ between the two cell lines MCF7 and T47D (rs12247456: AA *vs* GG; rs2435357: CC *vs* TT), possibly accounting for their different *RET* expression patterns. SNP rs2435357 (also known as RET+3 polymorphism) is located at +5 kb inside intron 1 and shown to be associated with reduced expression of the gene, as *in vitro* assays have demonstrated that the T variant disrupts a SOX10 binding site within a highly conserved sequence (MCS+9.7) thus compromising *RET* transactivation [23,27]. Consistent with these data, T47D cells carry the genotype associated with lower expression of the *RET* gene, an observation which might explain both the reduced expression of the *RET* gene and the remarkable response to Nabut, different from the MCF7 response.

**Figure 8:**
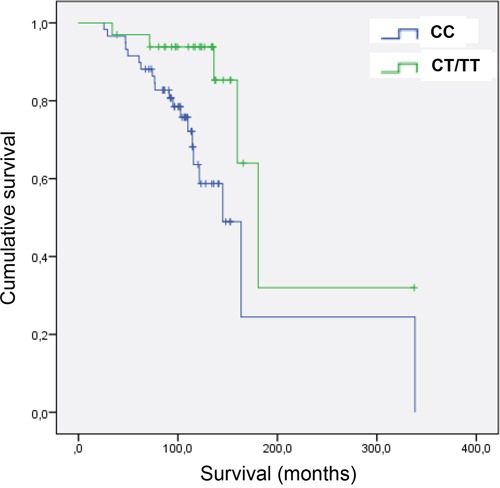
Overall survival (OS) in BC patients carrying the variant allele of SNP rs2435357 The two ROC curves show increased overall survival (OS) in carriers of the variant T allele of SNP rs2435357, among a pilot cohort of 93 ER+ breast cancer patients.

To verify whether the different alleles have effects *in vivo*, the rs2435357C>T SNP was genotyped in a cohort of 93 ER + BC patients (see Table 2 for clinical description of the patients). Intriguingly enough, the presence of at least one variant allele (CT or TT) was associated with an increased Overall Survival (OS) compared to patients carrying the CC wild type alleles. In particular, patients carrying the CC genotype displayed a median OS of 145.1 months (95% CI: 115.6 - 338.3) while those carrying the CT/TT genotype had a median OS of 180.6 months (95% CI: 159.7 - not estimable). The association was confirmed in multivariate analyses, where nodal status, grading, HER2 status and Ki67 were adjusted for (HR=0.243, 95%; CI=0.088-0.675; P=0,007) (Figure 8). This result is fully consistent with the observation that *RET* over-expression is associated with poor prognosis in ER+ BC and strongly suggests this SNP as prognostic factor in this subset of patients.

**Table 2:** Clinical parameters of 93 patients affected by breast cancer

	N (%)
**Age** (years) median, range	70 (50-92)
**Adjuvant CT** Yes No	44 (47.3) 49 (52.7)
**Adjuvant HT** Yes No	89 (95.7) 4 (4.3)
**Type of HT (N=89)** TAM AI TAM-AI	13 (14.6) 56 (62.9) 20 (22.5)
**Nodal Status** Negative Positive Unknown	50 (53.8) 42 (45.1) 1 (1.1)
**HER2 status** Positive Negative Unknown	10 (10.8) 73 (78.4) 10 (10.8)
**Grading** G1 G2 G3 Unknown	32 (34.4) 46 (49.5) 9 (9.7) 6 (6.4)
**Ki-67** ≤ 20 > 20 Unknown	52 (55.9) 26 (28.0) 15 (16.1)

Abbreviations: CT= Chemotherapy. HT= Hormone therapy. TAM= Tamoxifen. AI= Aromatase Inhibitors

To further deepen into such an association, we sought to verify whether tumors assessed were *RET*-expressing and to compare *RET* levels in the breast cancer tissues of the different genotype groups. To this end, we collected 80 paraffin-embedded tumor tissues from BC patients already genotyped at the SNP rs2435357 locus. RNA extraction was followed by real-time PCR for all of them, as reported in the Supplementary Information file. Unfortunately, we could estimate the *RET* level only in 21 samples, namely those that returned assessable and reliable levels of the beta-2-microglobulin housekeeping, besides *RET*. Despite this was not enough to draw a genotype-phenotype correlation, we could obtain *RET* expression data which overall confirmed the expression of this gene in the BC tissues collected (Supplementary Information file and Figure S3).

## DISCUSSION

The *RET* gene is a receptor tyrosine kinase which mediates the activation of intracellular pathways involved in proliferation, migration and differentiation of neural crest derived cells. It is regarded as the major oncogene involved in thyroid cancer, since *RET* rearrangements are detected in radiation-induced papillary thyroid carcinoma and gain-of-function mutations do segregate in inherited and sporadic medullary thyroid carcinoma [22].

More recently, *RET* has been involved in other forms of neoplasia including lung, pancreatic and breast cancers, thus demonstrating expanded oncogenic potentialities [22]. In particular, many studies have highlighted the role played by *RET* in estrogen receptor positive BC and its direct involvement in endocrine resistance [16]. Based on evidence collected so far, it is clear that *RET* involvement in BC is not due to mutations but to its over-expression. Moreover, *RET* is an estrogen inducible gene whose activation directly induces the estrogen receptor pathway, which mediates resistance to endocrine treatment. Such an autoregulatory loop is intriguing and has to be taken into consideration for the development of new targeted therapies in BC [45].

After confirming the expression of *RET* and of its ligands and coreceptors in two BC cell lines, MCF7 and T47D, here we have sought to investigate the possible molecular mechanisms underlying the different *RET* levels of these cells. We firstly established that the differences observed are mainly due to transcriptional events and not to DNA rearrangements. Given that the mean estimated half life of human transcripts is 3 hrs, and mRNA species with short half-life are enriched among genes with regulatory functions [52], we would have expected *RET* mRNA to be much more unstable and to start to decay earlier than 6-12 hrs. Therefore, the results obtained in our experiments are enough to conclude that the peculiar *RET* transcript stability observed in the BC cell lines tested might be the underlying cause of their sustained *RET* expression level. Thus, we investigated the effect of estrogens on *RET* expression, confirming that in both cell lines *RET* is estrogen inducible and interestingly finding that estrogens do modulate the expression of the two gene isoforms, *RET9* and *RET51*. Several studies have highlighted a different role for *RET9* and *RET51*, leading to the conclusion that *RET51* activates a specific oncogenic pathway [21]. Here we have shown that, consistently with previous observations, estrogens selectively up-regulate *RET51* compared to *RET9* [37] thus raising questions, deserving further investigations, about function of the two isoforms.

*RET* is also known to be expressed in subsets of immune cells and can orchestrate and activate the immune response in the gut [53,54]. In previous studies, we investigated the relationship between *RET* and the immune system finding that the activation of *RET-GDNF* pathway specifically modulates the expression of inflammatory cytokines and that *RET* expression is associated with *Interleukin 8* (*CXCl8* or *IL8*) transcript levels [48,55]. Here we report that *RET* expression in MCF7 and T47D is responsive to treatment with several inflammatory stimuli, besides *GDNF/gfra1* [48]. In BC cell lines, treatments with GDNF for 6 and 24 hours significantly reduced the amount of *RET* (negative loop) in MCF7 and at lesser extent in T47D, as previous studies had already shown [29]. The same reduction was observed in MCF7 after treatment with IL8 and TNFα, and a similar trend was also observed in T47D. These data suggest that *RET*-expressing BC cells still maintain sensitivity to cytokines, such as IL8 and TNFα, which are physiologically produced by the innate system to contrast tumor development. Moreover, a significant upregulation of IL-8 after 6 hrs of treatment with *GDNF/gfra1* in the MCF7 cells could be shown, a circumstance which suggests that RET activation mediates a fast IL-8 production in these cells, giving rise to a loop where RET activation seems to enhance the inflammatory effects of cytokine secretion.

*RET* over-expression is considered as a negative prognostic factor in ER+ tumors and significantly associated with development of endocrine resistance. Looking for molecules able to modulate *RET* expression, we focused on deacetylase inhibitors such as Nabut and deAza, previously investigated by us and known to modulate *RET* expression [39]. Deacetylase inhibitors are known to have different effect on estrogen dependent genes in BC cell lines. In ER- cells this class of compounds reactivates *ER* and ER-dependent target gene expression. Conversely, in ER+ cells deacetylase inhibitors have the effect of decreasing the expression of estrogen receptor and of its target genes. In our previous work, we demonstrated for the first time that Nabut but not deAza can reactivate *RET* expression in cells expressing low levels of *RET* such as lymphoblasts and neuroblastoma SKNMC cells, while it had no effect on cells expressing high levels of *RET* such as the MTC-TT line [39]. Our present experiments have shown that the two estrogen receptor expressing BC cell lines, MCF7 and T47D, have opposite *RET* reactions when treated with increasing doses of Nabut. These results have prompted us to characterize the genotype of the two BC cell lines, looking for ERE candidate sequences and known *RET* enhancers. Interestingly, we have found that T47D cells, which express less *RET* mRNA than MCF7 cells, are homozygous for the T allele of SNP rs2435357, already described to be associated with a reduced expression of *RET* [27].

In the light of this observation, we have tested the hypothesis that the presence of the variant allele of SNP rs2435357, assuring low *RET* levels, can be found in association with either less severe oncogenic phenotypes or a set of more favorable prognostic parameters. Consistent with our expectation, the genetic analysis of 93 ER+ BC patients (4 patients never started adjuvant endocrine therapy) at the SNP rs2435357 (C>T) locus has shown that the presence of at least one variant allele (genotypes CT and TT) is associated with an increased OS compared to patients carrying the CC wild type allele. Such an association has been confirmed in multivariate analyses, where nodal status, grading, *HER2* status and Ki67 were adjusted for.

The down-regulation of the RET gene achieved through the variant allele of SNP rs2435357 is clearly the reason for the observed OS increase. The same SNP is known to be associated with Hirschsprung disease (HSCR) through a similar mechanism: lack of adequate levels of *RET* expression during development does predispose to impaired enteric nervous system and therefore to colonic aganglionosis [18]. Very recently another RET SNP, rs2506030, located about 125 kb upstream of *RET*, has been reported as the second largest known genetic risk factor in HSCR [56]. These two *RET* SNPs, rs2435357 and rs2506030, have very low linkage disequilibrium in European-ancestry controls thereby suggesting two independent, and therefore additive, genetic effects at *RET*. Thus, it may be worthwhile to extend the genetic analysis to this second *RET* SNP in ER+ BC patients. Moreover, given the increasing number of cancers turned out to be associated with either rearranged or mutated forms of the *RET* receptor or *RET* gene over-expression, studies similar to that presented here might be fruitfully carried out in patient sets affected with different *RET*-related neoplasia.

The association reported here between *RET* SNP rs2435357 and OS in ER+ BC patients is of utmost importance and fully consistent with the observation that *RET* over-expression is associated with poor prognosis in ER+ BC and strongly candidate this SNP as prognostic factor in ER+ breast cancer. In addition, our findings suggest that *RET* and its downstream pathway can be proposed as a therapeutic target in order to improve response to endocrine therapy in selected BC patients. Indeed, targeting the *RET* tyrosine kinase activity is already an active line of research for the development of therapies against other *RET*-related tumors such as Medullary Thyroid Carcinoma [57].

## MATERIALS AND METHODS

### Cell lines and treatments

MCF7 and T47D cell lines were grown in Dulbecco's modified Eagle's medium (DMEM) with 10% FCS and 1% L-Glutamine 100X, 100U/ml penicillin and 100 μg/ml streptomycin. 17β-estradiol 1nM, Sodium Butyrate (Nabut) 1, 2, 5nM and 5 aza-2′ deoxycytidine (deAza) 10uM were used for treatments. Moreover, cells were also stimulated with either human GDNF and human GFRα1 at the final concentration of 100 ng/ml and 1μg/ml respectively (R&D Systems Minneapolis, MN, USA), TNFα (R&D System Minneapolis, MN, USA) 40 ng/ml, or IL8 (R&D System Minneapolis, MN, USA) 10 ng/ml. Cells were then incubated at 37°C in 5% CO^2^ and harvested at 6 and 24 hours after treatment.

### RNA isolation and Real Time PCR

Total RNA from cells was isolated by a commercial RNA purification kit (RNeasyMini kit, Qiagen, GmbH, Germany) according to the manufacturer's protocol. One μg of total RNA was reverse transcribed with iScript cDNA Syntesis kit (Bio-Rad Hercules, CA, USA) according to the manufacture's protocol. Real-time quantitative PCR was performed using inventoried Assays-on-Demand™ provided by Applied Biosystem. Hs01120027_m1 was used to detect the *RET* gene and Hs99999903_m1 was used to detect the reference gene *Beta-Actin*. mRNA half-life was detected after treatment with 5,6-Dichloro-1-Beta-D-ribofuranosylbenzimidazole (DRB), an inhibitor of transcription [58]. To study ligands and coreceptors of *RET* we used Assays-on-Demand™ for *GDNF* (Hs00181185_m1), *NRTN* (Hs00177922_m1), *ARTN* (Hs00365083_m1), *PSPN* (Hs00358822_g1), *GFRα1* (Hs00237133_m1), *GFRα2* (Hs00176393_m1), *GFRα3* (Hs00181751_m1). PCR reactions were performed using the iQ™5 Real Time thermal cycler (Bio-Rad Hercules, CA, USA). The expression of mRNA was evaluated using the relative Ct method (ΔΔCt) and Real Time PCR amplification was performed in triplicate and repeated at least twice. To detect the two *RET9* and *RET51* isoforms, the same forward primer 5′-CGT CCA CTC CAT CTG ACT CC-3′ was used together with reverse primers 5′-GAT AGT GCA AAG GGG ACA GC-3′ for *RET9* and 5′TAG TGC CAT CAG CTC TCG TG-3′ for *RET51*. *β*_*2*_*microglobulin* was used as reference gene (Forward: 5′-AGG ACA AGA AGC CCT GAG CA-3′; Reverse 5′-GCCGTC TTC CCC TCC ATC-3′). The expression of mRNA was evaluated using the relative Ct method (ΔΔCt) and the Pfaffl method when the efficiencies of the amplicons were not similar. Genomic *RET* content was quantified by using primers in 5′ and 3′UTR of the gene described in Griseri et al. [59].

### Sequencing and patients analysis

DNA samples from 93 patients affected by ER+ BC were collected in the Laboratory of Cancer Genetics and Translational Oncology, in Cuneo. The study was approved by the Ethical Committee of the Croce & Carle Teaching Hospital in Cuneo, and patients recruited upon signing a specific Informed Consent. Genomic DNA was extracted from peripheral lymphocytes by a standard technique and subjected to *RET* SNPs screening by means of direct sequencing of the corresponding amplification products. PCR products were purified by ExoSAP IT (GE Healthcare) and directly sequenced using Big Dye v1.1 and a ABI3130 automated sequencer (Applied Biosystems, Foster City, CA, USA). Genetic screening for SNP rs2435357 (C>T) was performed by using primers F: 5′-AGAGGCACCAGGGTCAAAG-3′ and R: 5′-ATGCAAAGGAAACTGCCAAT-3′. Additional PCR details are available upon request.

Overall Survival (OS) was defined as the time between surgery and death, whatever the cause. Observation time of patients alive at the last follow-up was censored. Confidence intervals of median survival times were calculated according to the log–log method of Brookmeyer and Crowley. Hazard ratios and appropriate 95% CIs were calculated by means of the Cox proportional hazard model. A multivariate Cox regression model was fitted to evaluate the independent effect of rs2435357 on OS. Starting from a full model, including all covariates, non significant variables were progressively removed according to a backward stepwise procedure based on the Wald's chi-square test.

## SUPPLEMENTARY DATA FIGURES


