# Supplementary material for: Genetic and epigenetic factors affect RET gene expression in breast cancer cell lines and influence survival in patients
Source: Oncotarget. 2016 Mar 28;7(18):26465–79. doi: 10.18632/oncotarget.8417 (PMC5041993; doi:10.18632/oncotarget.8417)
Supplement: Supplementary file 1 [file oncotarget-07-26465-s001.pdf]

SUPPLEMENTARY DATA

MATERIALS AND METHODS

Quantitative Real Time PCR (qRT-PCR): long term RET mRNA stability

mRNA half-life was detected in the T47D cell line after 6 hrs and 12 hrs treatments with 5,6-Dichloro-1-Beta-D-ribofuranosylbenzimidazole (DRB), an inhibitor of transcription. Total RNA from cells was isolated by a commercial RNA purification kit (RNeasyMini kit, Qiagen, GmbH, Germany) according to the manufacturer's protocol. One µg of total RNA was reverse transcribed with iScript cDNA Syntesis kit (Bio-Rad Hercules, CA, USA) according to the manufacture's protocol. Real-time quantitative PCR was performed using inventoried Assays-on-Demand™ (Thermo Fisher Scientific, Waltham, MA USA) Hs01120027_m1 to detect the *RET* gene and Hs99999903_m1 to detect the reference gene *Beta-Actin*. PCR reactions were performed using the iQ^{TM5} Real Time PCR (Bio-Rad Hercules, CA, USA). The expression of mRNA was evaluated using the relative Ct method (ΔΔCt) and Real Time PCR amplification was performed in triplicate and repeated at least twice.

Quantitative Real Time PCR (qRT-PCR): IL-8 expression in response to RET activation

MCF7 and T47D cell lines were grown as reported in the full paper and stimulated with human GDNF and human GFRα1 at the final concentration of 100 ng\ml and 1µg\ml respectively (R&D Systems Minneapolis, MN, USA). Cells were then incubated at 37°C in 5% CO_2 and harvested at 6 and 24 hours after treatment. Total RNA was isolated by a commercial RNA purification kit (RNeasyMini kit, Qiagen, GmbH, Germany) according to the manufacturer's protocol. One µg of total RNA was reverse transcribed with iScript cDNA Syntesis kit (Bio-Rad) according to the manufacture's protocol. Real-time quantitative PCR was performed using inventoried Assays-on-Demand™ (Applied Biosystem). The expression of IL-8 receptor, *CXCR2*, was studied with the assay Hs00174304_m1. PCR reactions were performed using the iQ^{TM5} Real Time PCR (Bio-Rad Laboratories). The expression of mRNA was evaluated using the relative Ct method (ΔΔCt) and Real Time PCR amplification was performed in triplicate and repeated at least twice.

Quantitative Real Time PCR (qRT-PCR): RET expression in patients' BC cells

Total RNA was obtained from paraffin-embedded samples of different patients using RecoverAll™ Total Nucleic Acid Isolation Kit for FFPE (ThermoFisher Waltham, MA USA #1975). 500 ngs of extracted RNA were reverse transcribed with iScript cDNA Synthesis kit (BioRad #170-8891) according to the manufacture's protocol. Quantitative Real-Time PCR was performed using inventoried Assays-on-Demand™ provided by Applied Biosystem. TaqMan probe human Hs01120027_m1was used to detect the *RET* gene and Hs99999907_m1 was used to detect the reference human gene *B2M*. PCR reactions were performed using the iQ^{TM5} Real Time PCR (Bio-Rad Laboratories). The mRNA expression was evaluated using the relative Ct method (ΔΔCt) and Real Time PCR amplification was performed in triplicate and repeated at least twice.

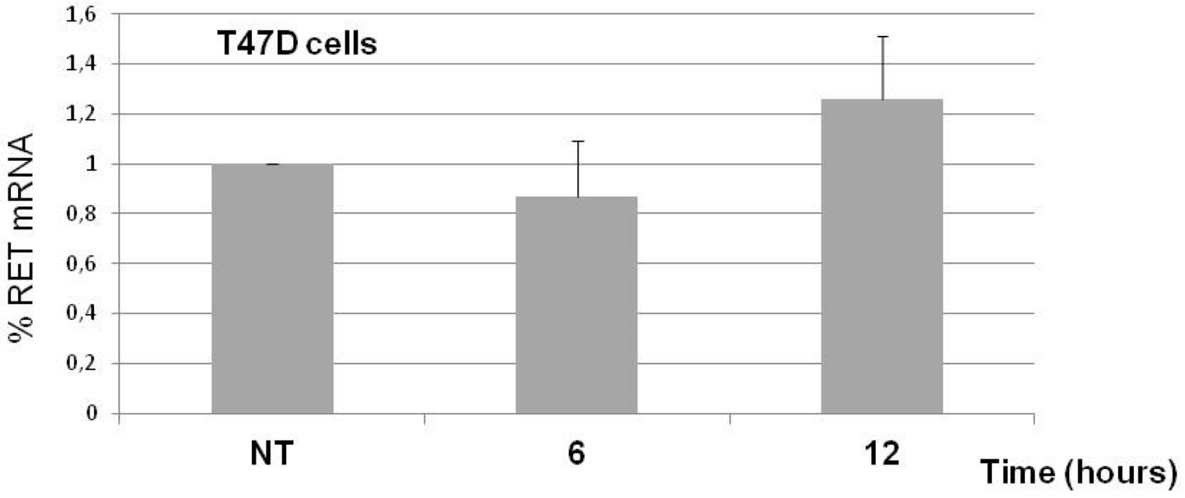

Supplementary Figure S1: Quantification of *RET* mRNA stability after 6 and 24 hours of DRB treatments in T47D cells. Values are the mean ±SD of at least two independent experiments performed in triplicate. No difference between the three conditions has resulted significant (*p>0.05*).

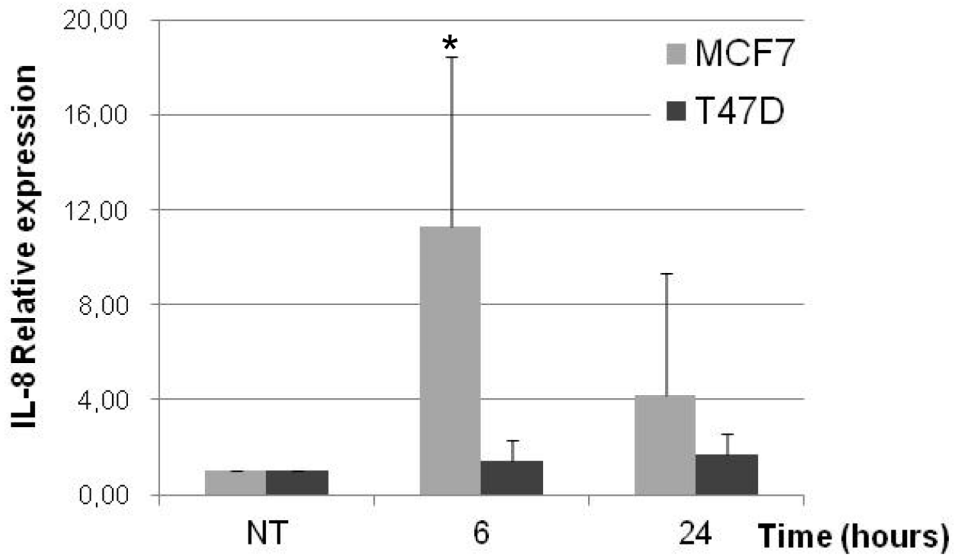

Supplementary Figure S2: ***IL-8*** **expression in MCF7 and T47D cells treated for 6 and 24 hours with GDNF/GRFα1.** Values are the mean ±SD of at least two independent experiments performed in triplicate; asterisks (*) indicate statistically significant differences *(*p<0.05)*.

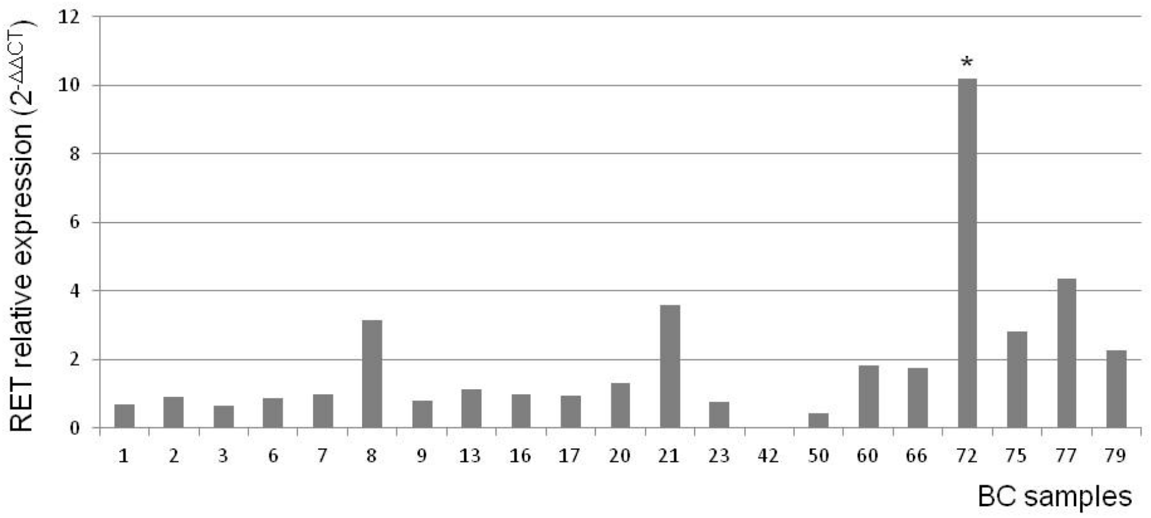

Supplementary Figure S3: *RET* relative expression levels (fold changes over the mean expression of all the samples) estimated in RNA extracted from tumor tissues of BC patients. Values are the mean of at least two independent experiments performed in triplicate. The asterisk indicates a statistically significant outlier level of expression, as assessed after performing the Grubbs' test.
